# Editorial: Archaea in the Environment: Views on Archaeal Distribution, Activity, and Biogeography

**DOI:** 10.3389/fmicb.2021.667596

**Published:** 2021-03-12

**Authors:** Andreas Teske, Ricardo Amils, Gustavo A. Ramírez, Anna-Louise Reysenbach

**Affiliations:** ^1^Department of Marine Sciences, University of North Carolina at Chapel Hill, Chapel Hill, NC, United States; ^2^Planetology and Habitability Department, Centro de Astrobiología, Madrid, Spain; ^3^Western University of Health Sciences, College of Veterinary Medicine, Pomona, CA, United States; ^4^Biology Department, Portland State University, Portland, OR, United States

**Keywords:** archaea, diversity, habitat, ecology, activity, environment

On the occasion of the 10-year anniversary of Frontiers in Microbiology, this Research Topic was launched to highlight the linkages between extreme and archaeal microbiology (Teske, 2020). Archaea adapt to the physical and chemical characteristics of their habitat—such as organic matter availability, electron donor redox status, salinity, temperature, and pH—in terms of metabolic activity, community composition, gene expression patterns, and evolutionary diversification (Baker et al., 2020). Here, cultivation- and genome-based studies highlight linkages between archaea and their habitats (Figure 1).

**Figure 1:**
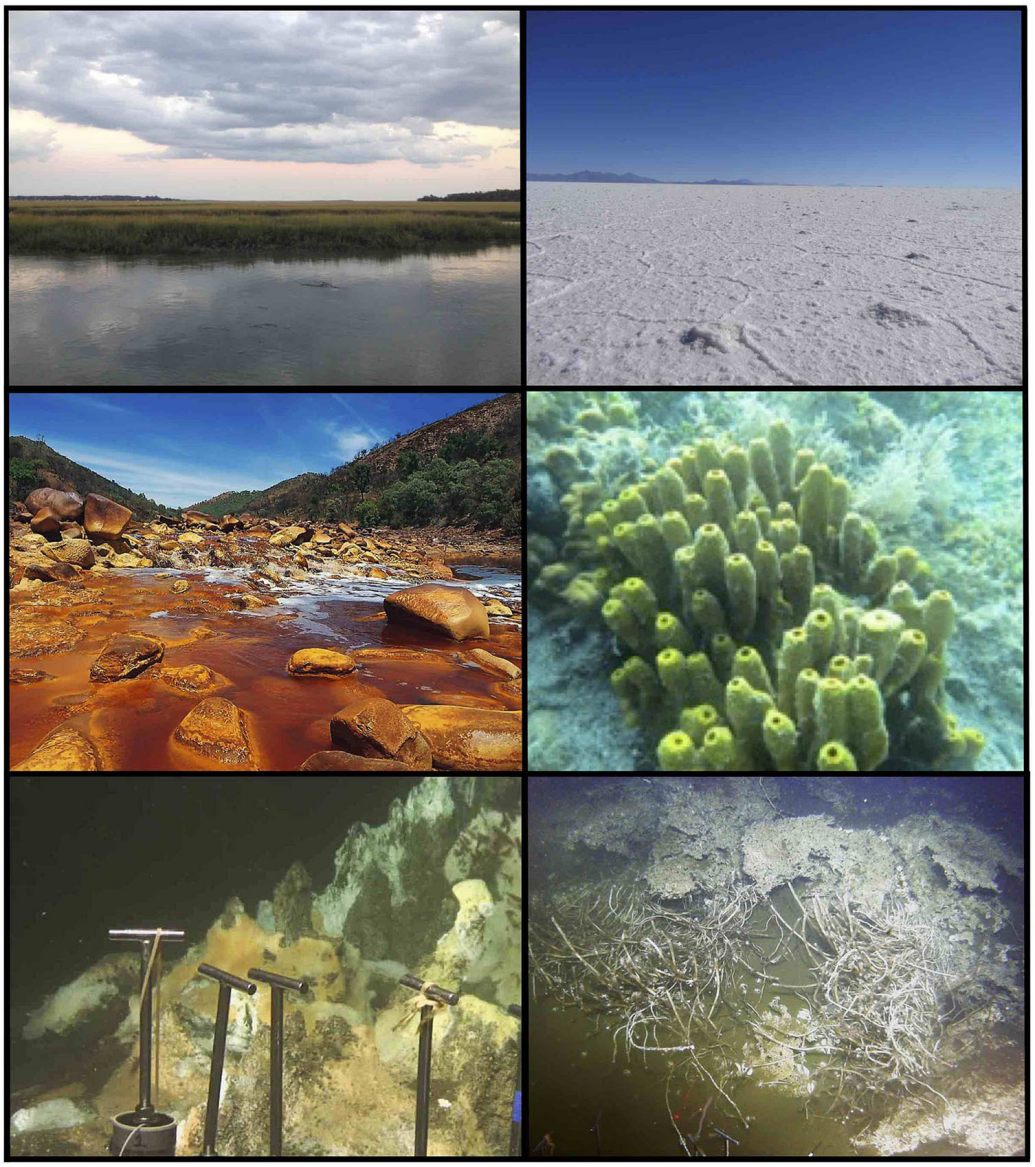
Diverse archaeal habitats. Top row, the archaea-rich White Oak River estuary in North Carolina, USA (left), and the Salar de Uyuni, the world's largest salt flat and habitat for extremely halophilic archaea in the Andean Altiplano, Bolivia (right). Center row, the well-studied AMD model system of the acidic and metal-rich Rio Tinto, Spain (left), and the thaumarchaeotal symbiont-bearing sponge *Aplysina aerophoba*, Mediterranean Sea (right). Bottom row, hydrocarbon-rich hydrothermal sediments with microbial mat overgrowth (left), and cold seep carbonates and tubeworms in Guaymas Basin, Mexico (right). Photographs courtesy of D. Steen, N. Rodríguez, J. Segura, Steindler Lab, and WHOI Alvin group, respectively.

An important question is the variability of archaeal genotypes and phenotypes within specific lineages at the genus- or family level. This variability defines the limits of adaptation for an archaeal group and therefore delineates its environmental range. In a study of physiological and genomic diversity within cultured isolates and high-quality environmental genomes of the extremely acidophilic, metal-and sulfur-oxidizing, facultatively autotrophic genus *Metallosphaera* (Wang P. et al.), similarity in carbon fixation, sulfur assimilation, nitrate reduction, and sulfur- and iron-oxidizing capabilities coexisted with species-specific variations in specific pathways. The evolving physiological and phylogenetic diversification within *Metallosphaera* is shaped by competing genome expansion *via* horizontal gene transfer to acquire new capabilities, and by adaptive genomic streamlining and gene loss.

As comparative genomic studies highlight ecophysiologically relevant differences among related cultured archaea, comparative field surveys of uncultured archaea infer ecophysiological traits from habitat preferences. A sequence-based archaeal survey of Thermoplasmatales-dominated metal-rich and low-pH sediments from an acid mine drainage (AMD) stream in Wales (Distaso et al.) showed that patterns of family- and genus-level uncultured lineages from this site differed from other well-studied AMD sites with distinct pH, redox status and metal and organic matter content, such as the Rio Tinto in southern Spain, Iron Mountain in California, and Carnoules Mine in France. Comparative analyses will gain ecophysiological and taxonomic specificity once more acidophilic lineages within the Thermoplasmatales are brought into culture, something that has been achieved for bacterial AMD community members to a far greater degree.

In part due to increasingly comprehensive global surveys (Nayfach et al., 2020), the archaeal domain is no longer regarded as a collection of extremophiles that inhabit niche environments. For example, archaeal communities thrive along the path of freshwater flow from upland soil to the sea, starting with agricultural soil, continuing with freshwater biofilms and concluding with estuarine waters (Wang H. et al.). The ammonia-oxidizing Thaumarchaeota are conspicuously present in all three environments, and their diversity gradient reflects an overall trend of greatest archaeal diversity in soil (Nitrososphaerales, Nitrosotaleales, and Nitrosopumilales), reduced diversity in freshwater biofilms (Nitrososphaerales) and lowest diversity in estuarine water (Nitrosopumilales). Within the Euryarchaota, the archaeal spectrum changes from the methanogenic Methanomasiliicoccales in soil toward other Methanomicrobia in freshwater biofilms and toward the Marine Group II (within the Thermoplasmatales) in estuarine water. These archaeal phyla have diversified and adapted successfully to colonize globally extensive, non-“extreme” habitats, and in doing so, affect global biogeochemical cycles, particularly of nitrogen and methane.

Among marine Thaumarchaeota, members of the ammonia-oxidizing, autotrophic Nitrosopumilales dominate the marine water column but also appear as endobionts in filter-feeding sponges (Hallam et al., 2006), where they metabolize ammonium and urea produced by their host. Haber et al. compared the genomes of thaumarchaeotal sponge symbionts to those of their free-living counterparts, and found genomic modifications overlap with bacterial sponge symbionts—enriched transposes, toxin-antitoxin and restriction modification systems. The presence of branched amino-acid transporters, not found in free-living Nitrosopumilales, indicates heterotrophic amino acid metabolism and therefore a mixotrophic metabolism. This study provides an excellent example showing how archaea evolve and diversify not only as free-living microorganisms in response to environmental characteristics, but also as obligate symbionts in intimate associations with particular host species, as shown by host-specific genomic adaptions.

In some cases, the biochemistry of a particular substrate uptake and assimilation process limits the environmental competitiveness of a physiological class of archaea. Wormald et al. examine acetoclastic and hydrogenotrophic methanogenesis in alkaline soils and sediments and find that, above pH 9, hydrogenotrophic methanogenesis dominates and acetoclastic methanogenesis is no longer active; acetate continues to be utilized but only *via* syntrophic acetate fermentation with H2-utilizing methanogenic partners. Since acetate occurs as the acetate anion under alkaline conditions, the authors conclude that the energy expense for transmembrane anion uptake renders otherwise exergonic acetoclastic methanogenesis non-competitive. However, hydrogenotrophic, acetoclastic and methylotrophic methanogenesis remain detectable in serpentinization-derived alkaline fluids with pH up to 11 (Crespo-Medina et al., 2017), indicating that additional parameters are shaping the environmental persistence of methanogenic pathways.

Examples for bioprospecting of enzymes from extremophilic archaea are based on isolations of obligately halophilic, photoheterotrophic archaea from hypersaline salt lakes and salterns in India: Haloarchaeal bacteriorhodopsins and V-type ATPases from *Haloarcula, Halomicrobium*, and *Haloferax* isolates (Verma, Chaudhary, et al.), and an alpha-amylase from isolates related to *Halogeometricum borinquense* and *Haloferax volcanii* (Verma, Vasudeva, et al.). In contrast to pre-genomic studies, the enzymatic characterizations of these recombinant bacteriorhodopsins and alpha-amylase are supplemented by whole-genome analyses of the source organisms, to identify a fuller spectrum of candidate enzymes and their distribution across different halophilic taxa.

The final contribution of this Research Topic (Teske et al.) focuses on bacterial and archaeal communities in surficial hydrothermal sediments and recently discovered cold seeps of Guaymas Basin (Figure 1). Methane-cycling archaea are strongly affected by thermal regime in underlying sediments, whereas heterotrophic or mixotrophic sediment archaea (Thermoplasmata, Bathyarchaeota, and Lokiarchaeota), hyperthermophiles, and sometimes Thaumarchaeota coexist in the ecotone of temperate surficial sediments. Genomic exploration of surficial Guaymas sediments is yielding numerous novel archaeal lineages (Dombrowski et al., 2018) and is expanding the archaeal domain.

To conclude, archaeal culture- and genome-based findings are continuously providing new insights into the physiological adaptability and environmental range of archaea, and ultimately provide context to understand the evolutionary diversification of the archaeal domain throughout the microbial biosphere.

## Author Contributions

AT wrote the Editorial. All authors edited and commented on it.

## Conflict of Interest

The authors declare that the research was conducted in the absence of any commercial or financial relationships that could be construed as a potential conflict of interest.
